# Combining Visible Light and Non-Focused Ultrasound Significantly Reduces *Propionibacterium acnes* Biofilm While Having Limited Effect on Host Cells

**DOI:** 10.3390/microorganisms9050929

**Published:** 2021-04-26

**Authors:** Mark E. Schafer, Tessie McNeely

**Affiliations:** 1 School of Biomedical Engineering, Science and Health Systems, Drexel University, Philadelphia, PA 19104, USA; 2 Photosonix Medical, Inc., Fort Washington, PA 19034, USA; tmcneely@photosonixmed.com

**Keywords:** biofilm, ultrasound, blue light, *Propionibacterium acnes*, bacteria, antibacterial

## Abstract

Bacterial biofilms are highly resistant to antibiotics and have been implicated in the etiology of 60%–80% of chronic microbial infections. We tested a novel combination of low intensity ultrasound and blue light against biofilm and planktonic bacteria. A laboratory prototype was built which produced both energies uniformly and coincidently from a single treatment head, impinging upon a 4.45 cm^2^ target. To demonstrate proof of concept, *Propionibacterium acnes* biofilms were cultured on Millicell hanging inserts in 6-well plates. Hanging inserts with biofilms were treated in a custom exposure chamber designed to minimize unwanted ultrasound reflections. Coincident delivery of both energies demonstrated synergy over either alone, killing both stationary planktonic and biofilm cultures of *P. acnes*. Reduction in biofilm bacteria was dose dependent on exposure time (i.e., energy delivered). *P. acnes* biofilms were significantly reduced by dual energy treatment (*p* < 0.0001), with a >1 log_10_ reduction after a 5 min (9 J/cm^2^) and >3 log_10_ reduction after a 30 min (54 J/cm^2^) treatment (*p* < 0.05). Mammalian cells were found to be unaffected by the treatment. Both the light and the ultrasound energies are at levels previously cleared by the FDA. Therefore, this combination treatment could be used as a safe, efficacious method to treat biofilm related syndromes.

## 1. Introduction

Bacteria in biofilms have a dramatically altered phenotype compared to planktonic bacteria with respect to growth rate and gene transcription. The biofilm phenotype also differs from that of purely sessile cells, such as may be seen growing on agar plates [1]. Many genes necessary for planktonic metabolism are turned off, since the cells in a biofilm are not rapidly dividing [2,3]. Other genes responsible for the biofilm phenotype are upregulated. Due, in part, to the damping down of their metabolism, biofilm encased bacteria are highly resistant to the effects of antibiotics, biocides and other drugs which are used to eliminate planktonic bacteria [2]. Chronic use of antibiotics also leads to drug resistance. Thus, when considering development of new treatment approaches, there are justifiable reasons to consider treatments that do not rely on drug entities.

One such alternate approach is the use of various types of energy to generate bactericidal activity, including light and ultrasound. Planktonic bacteria are susceptible to killing by light in the blue/violet spectrum (400–470 nm) [4,5]. Although the exact molecular mechanism is not precisely known, the bactericidal effect of blue light involves excitation of porphyrins or other chromophores within the bacteria, which produces toxic oxygen products [6,7,8]. In an environment with low levels of free iron, such as found in human tissue, conversion of porphyrin precursors to heme is restricted, thus leading to an excess of porphyrins in the bacteria [9,10]. Porphyrin photoexcitation occurs maximally within the Soret band (360–460 nm), with four smaller peaks between 500 and 635 nm (the green to red range) [9,10]. In the presence of oxygen, photosensitized porphyrins produce singlet oxygen and hydroxyl free radicals, which are highly toxic to the cell [7,11].

Low intensity ultrasound has been explored for its action on planktonic bacteria and biofilms. Although it does not have direct bactericidal activity, it may induce physical or metabolic intracellular changes so that biofilms become susceptible to killing by biocides, antibiotics, or other reagents. The exact mechanism of action for the low intensity ultrasound effect on cells has not been fully established, but it may involve mechanical deformation of the cellular membranes, leading to increased metabolic activity and oxygen influx [12,13]. There is mounting evidence that the effects of low-dose ultrasound include increased permeability of cell membranes [12,14]. Ultrasound perturbs the cell membrane and stimulates active uptake, or permits passive uptake, through a temporary disruption of the membrane or other structural cell component [13,15]. Thus, after application of low intensity ultrasound, cells in biofilms become susceptible to bioactive molecules, including antibiotics [16,17,18]. Ultrasound additionally induces an oxidative stress response through deformation of cell membranes, with subsequent influx of oxygen, and induction of NO production [19,20]. NO at low concentrations is a trigger for bacteria phenotype transition, leading to dispersal of bacteria from a biofilm [21,22,23].

With an appreciation of the individual effects of light and ultrasound, it was postulated that the combination of the two would synergize and promote bacteria killing and biofilm elimination. A device delivering both energies from the same treatment head to the same tissue volume was therefore developed. The combined energy was characterized for its bactericidal activity. To explore the potential of using this technology to treat dermal infections, the skin commensal *Propionibacteria acnes* was chosen for investigation. *P. acnes* plays a pivotal role in the pathology of acne vulgaris, and can be found growing as biofilms in situ, within follicles in sebum rich areas of the face and back [24,25]. To investigate potential “off target” effects on host tissue, two mammalian cells lines were investigated for gross damage after combined energy exposure.

## 2. Materials and Methods

### 2.1. Preparation of Bacteria Stocks

*P. acnes* strain 6919 (ATCC 6919) [26,27] was selected for biofilm production [24,28,29]. *P. acnes* bacteria were cultured 4 days on Brucella 5% sheep blood agar (Remel) at 37 °C under anaerobic conditions using BD GasPaks, EZ Gas Generating Pouch systems (VWR). Bacteria were harvested from plates, pelleted, resuspended in phosphate buffered saline (PBS) containing 16% glycerol, and stored in 1 mL aliquots at −20 °C. All experiments were performed starting with a frozen aliquot.

### 2.2. Biofilm Growth Optimization

Bacteria culture conditions were investigated for enhanced growth on Millicell^®^ hanging inserts with a polyethylene terephthalate (PET) membrane (4.45 cm^2^, 0.4 µm, PIHT30R48, EMD Millipore, Burlington, MA), in 6-well culture plates (EMD Millipore PIMWS0650). The following steps were optimized: culture medium for bacteria adherence to PET membrane, seeding density, time for cell adherence, culture medium for continued growth, and time of harvest. (Table 1). Presence of uniform biofilms was confirmed by staining with Congo red (Fisher S25264), crystal violet, and Live/Dead BacLight reagents (Fisher L7012) (data not shown).

### 2.3. Biofilm Preparation for Experiments

Frozen aliquots of bacteria were thawed and diluted in adhering medium and 2 mL of suspension inoculated into hanging inserts. Inserts were placed in 6-well plates containing 4 mL adhering medium/well and incubated at 37 °C (without shaking) in BD anaerobic GasPaks™ (BD, Franklin Lakes, NJ). After incubation to allow for adherence, inserts with adhered bacteria were moved to fresh 6-well plates, refreshed with growth medium (as indicated in Table 1), and incubated for varying times at 37 °C. For the energy exposure experiments, inserts were removed from the plates and rinsed firmly with 4 × 0.5 mL sterile normal saline to remove loosely adhered cells, cell aggregates, or loosely adhered biofilm. Remaining adherent biofilms were then ready for either energy exposure or biofilm staining. For staining and evaluation of biofilm viability, LIVE/DEAD BacLight was used.

### 2.4. Biofilm Exposure and Evaluation of Viable Bacteria

**Figure 1:**
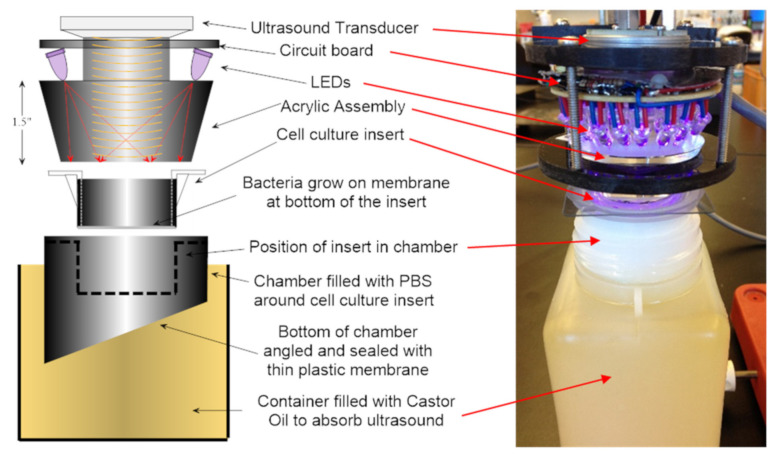
Experimental Setup (exploded view, left; photo, right).

Rinsed inserts with adhered biofilms were placed in the custom designed chamber containing 18 mL of sterile PBS (Figure 1). Approximately 7 mL of sterile saline was added inside the insert to conduct ultrasound and light to the biofilms. The surface of the acrylic cone, which emitted light plus ultrasound, was slightly submerged into the saline, and located 16 mm above the biofilms. After energy exposure, the insert was removed from the exposure chamber, and the PET membrane, to which the biofilms were attached, was cut from the insert using a sterile scalpel. The membrane was placed in a plastic test tube with 2 mL of PBS/0.2% Tween-80. Samples were vigorously vortexed and sonicated in an Elmasonic S-60H bath sonicator (Elma Schmidbauer, Singen, Germany) for 2 × 10 min at room temperature. Serial dilutions were made in PBS. The dilutions were plated onto reinforced clostridial agar (RCA, Oxoid, Hampshire, UK) plates, with 2 plates/dilution. For biofilm samples, data were reported as CFU per total 2 mL sample. Alternatively, for further incubation of the biofilms after exposure, the insert was returned to the 6-well plate with fresh growth medium. At time of harvest, the sample was processed as described above. For control, or mock exposures, the insert with sample was placed in the same exposure chamber as the test samples, but without light or ultrasound turned on. For ultrasound/light exposure of planktonic bacteria, *P. acnes* was cultured anaerobically for 4–5 days at 37 °C in RCM broth, pelleted, OD_600_ adjusted to 0.1 with saline, and 20 mL of suspension was added to the exposure chamber. Bacteria were exposed the same as the biofilm samples, and the treated suspension was collected for sonication, followed by serial dilution and plating.

### 2.5. Culture of Murine 3T3 and Human Primary Keratinocytes

Two mammalian cell types, murine fibroblasts (3T3) and human primary keratinocytes, were evaluated for susceptibility to the energy treatment. Murine 3T3 cells were maintained and passaged in DMEM containing L-glutamine (Gibco, Gaithersburg, MD), with 10% FCS (HyClone, Logan, UT), and penicillin (60 U/mL)/streptomycin (60 µg/mL). Human primary epidermal keratinocytes were obtained from the ATCC (PCS-200-011) and passaged and maintained in Dermal Basal Cell Medium (PCS-200-030) with Keratinocyte Growth kit (PCS-200-040) factors, as per the ATCC directions. All cells were cultured at 37 °C in a 5% CO_2_ incubator in T75 tissue culture flasks. To prepare for experiments, cells were harvested from the T75 flasks and inoculated into transwell hanging inserts placed in 6-well plates, containing 1 mL medium in the insert and 3–4 mL medium in the well. For exposure experiments, cell cultures in hanging inserts were placed into the exposure chamber, with conditioned culture medium remaining within the insert. Sufficient additional culture medium was added to the insert to fill it to the top (approximately 6–7 mL). The exposure chamber was filled with DMEM (Gibco). The exposure chamber was maintained at 37 °C during exposures. Experiments were carried out on the bench top and, as such, sterility could not be maintained, although aseptic procedures were followed.

### 2.6. Evaluation of Mammalian Cell Viability

After energy exposure, cells in hanging inserts were analyzed for viability. Alamar blue (Invitrogen, DAL1025, Thermo Fisher Scientific, Waltham, MA) was added to the culture medium in the insert as per the manufacturer’s directions. After incubation at 37 °C, 5% CO_2_, 1 mL aliquots were removed, and absorbance was measured at 570/600 nm to determine the amount of alamar blue reduced by the mammalian cells—a measurement related to the metabolic activity of the cells. Since there was not a standard curve relating alamar blue reduction to viable cell number, cells were also assessed for viability using trypan blue. Cells were removed from the PET membrane with trypsin treatment using 0.25% trypsin/EDTA (HyClone, SH30042.01), followed by fetal calf serum (100%) (HyClone) to block the trypsin. For viability analysis, 50% trypan blue was added to cells prior to counting on a hemocytometer.

### 2.7. Energy Exposure System

**Figure 2:**
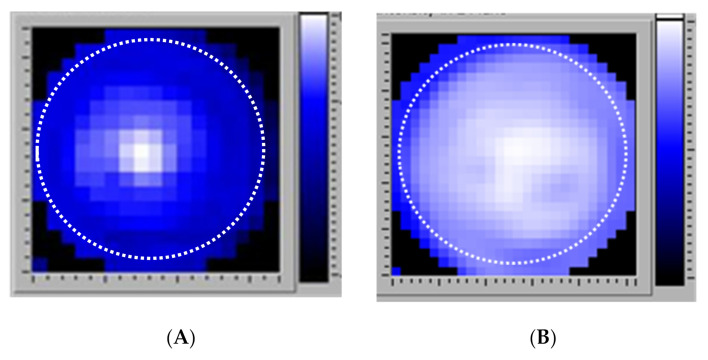
Ultrasound pressure distribution pattern before (**A**) and after (**B**) modifications. Dotted circles indicate the diameter of the hanging insert.

The laboratory exposure system (Figure 1) was designed to treat the experimental samples with calibrated and repeatable levels of both light and ultrasound energy. An ultrasound transducer was coupled to the central region of the large end of an Acrylic^TM^ frustum (truncated cone). LEDs encircled the transducer and were aimed at the narrow end of the frustum to provide uniform light exposure across the target (i.e., hanging insert with adhered biofilm or mammalian cells, or a planktonic bacteria suspension). The ultrasound beam was designed to either insonify a central 16 mm^2^ zone or the entire surface of the insert (4.45 cm^2^). Figure 2 shows the ultrasound field coverage patterns, where white is indicative of maximum ultrasound pressure. The dashed lines represent the inner diameter of the insert. Unless otherwise noted, for experiments described herein, the 4.45 cm^2^ zone output was utilized. The system was calibrated using a custom ultrasound measurement system [30]. The 36 LEDs had a narrow band output at 405 nm, and a 4-channel pulse-width modulation light control was used, which permitted a 256:1 digital light level control via laptop computer. The light output was measured by a calibrated radiometer (ILT400, International Light Technologies, Peabody, MA). The overall system provided a wide range of output necessary to investigate the light/ultrasound interactions involved in biofilm treatment. Figure 1 shows the prototype device, as well as the custom exposure chamber in which the transwell was suspended for biofilm exposure. The energy parameters used to expose bacteria or mammalian cells are listed in Table 2.

### 2.8. Statistical Methods

Experiments were performed multiple times and representative data are shown. Statistical evaluation of CFU numbers or mammalian cell numbers was performed using GraphPad Prism v6.00 for Windows, and we chose one-way ANOVA for comparison of multiple means or a *t*-test for comparison of only two means.

## 3. Results

### 3.1. Synergistic Bactericidal Action Achieved by Combining Light Plus Low Intensity Non-Focused Ultrasound

**Figure 3:**
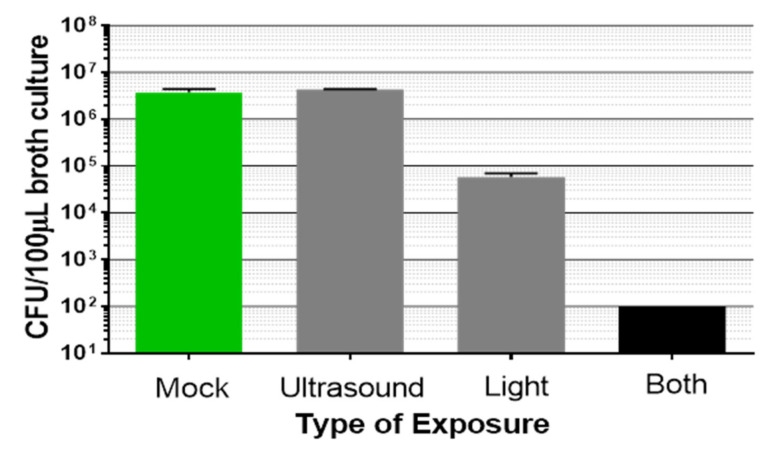
*P. acnes* planktonic culture (20 mL) exposed to either low intensity ultrasound, light at 405 nm, 80 mW/cm^2^ (96 J/cm^2^), or a combination of the two for 20 min, followed by serial dilution and plating of 100 µL on RCA for CFU enumeration. Bars are mean ± std. dev., n = 2, *p* = 0.0003 (ANOVA for differences of the means of the four determinations); *p* < 0.05 (*t*-test for differences of the means) comparing the combination treatment versus either light alone or ultrasound alone.

Using the custom-built laboratory exposure system, planktonic bacteria and biofilms were treated with either light or low intensity ultrasound, or both simultaneously. Planktonic bacteria (from a 4–5 days old stationary culture) were assessed first (Figure 3). Low intensity ultrasound did not induce observable bactericidal activity when planktonic bacteria were exposed for 20 min. Light at 405 nm was bactericidal, as previously reported by other investigators [31]. Importantly, in combination, the light plus low intensity ultrasound induced a synergistic bactericidal effect; that is, the reduction of bacteria CFU was greater than just adding the effects of the two single energy sources (*p* < 0.05).

**Figure 4:**
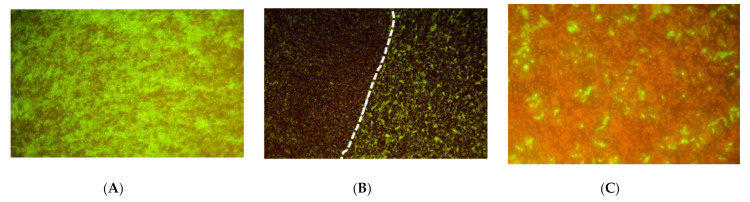
P. acnes biofilm: (**A**) Untreated control, magnification = 100×; (**B**) Exposure border. Biofilm exposed to low intensity ultrasound + light (left region, light @30 mW/cm^2^ for 30 min or 54 J/cm^2^) or light only (right region, light @ 30 mW/cm^2^ for 30 min or 54 J/cm^2^). Magnification = 10×; (**C**) Biofilm exposed to low intensity ultrasound + light (light @ 30 mW/cm^2^ for 30 min or 54 J/cm^2^). Magnification = 100×.

Next, biofilms were tested for sensitivity to the combination energy treatment. *P. acnes* biofilms were prepared as described in the Materials and Methods section, placed in the exposure chamber, and exposed to the combination of light and ultrasound. To directly examine the energy effect, biofilms were stained for the presence of viable cells using Live/Dead BacLight reagents and observed with a fluorescence microscope (Figure 4). In these experiments, therapeutic light exposure illuminated the entire biofilm surface (the total 4.45 cm^2^ membrane), but the ultrasound insonified only a 16 mm^2^ area centered in the light field (cf Figure 2, top). Therefore, biofilm reduction from the combination of the two energy sources could be distinguished from reduction by light alone. As seen in Figure 4A, there was a viable biofilm uniformly adhered to the PET membrane (green color indicates live cells). After exposure, as observed with lower magnification, the biofilm was qualitatively reduced where the two energy sources overlapped, compared to the light alone area, as indicated by the lack of green color (Figure 4B). With higher magnification (Figure 4C), it could be observed that bacteria in the overlap treatment area were directly killed by the simultaneous energy combination, where dead bacteria were stained red and living bacteria were stained green. Therefore, *P. acnes* biofilms were being killed by the combination treatment, which was not observed under these energy conditions in the light-alone treatment area.

**Figure 5:**
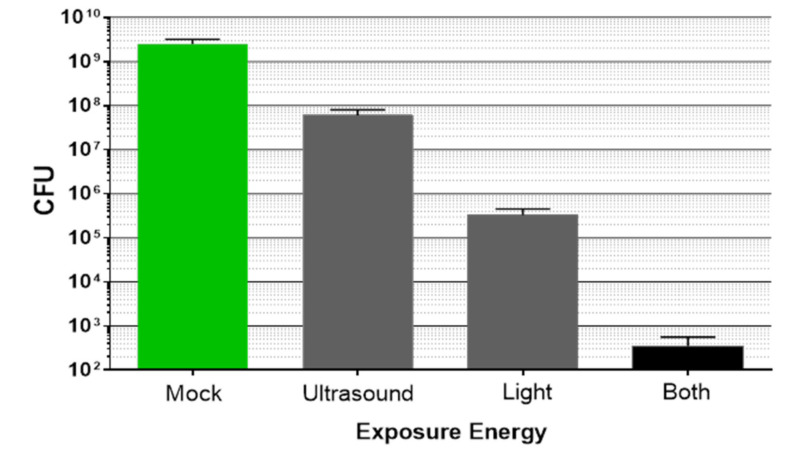
P. acnes biofilm exposed to either low intensity ultrasound 456 kHz@250 kPa, light at 405 nm, 80 mW/cm^2^ (144 J/cm^2^), or a combination of the two for 30 min, followed by harvesting of the biofilm and serial dilution and plating on RCA for CFU enumeration. Bars are the mean ± std. dev., n > 2, *p* = 0.0002 (ANOVA for differences of the mean of the four determinations); *p* < 0.05 (*t* test for differences of the means) comparing the combination treatment versus either light alone or ultrasound alone.

To quantify biofilm reduction after dual energy exposure, experiments were conducted in which the ultrasound field covered the entire transwell insert (4.45 cm^2^). After exposure, the biofilms were harvested and CFU enumerated. There was reduction of the biofilms by each energy source alone (Figure 5); however, combining the two energies resulted in a synergistic effect similar to that observed with planktonic bacteria. The combination treatment was significantly better than either individual energy source alone (*p* < 0.05).

In some experiments, the saline supernatant from the hanging inserts was collected after treatment to determine the number of surviving CFU released from the PET membrane during treatment. CFU recovered in the saline after dual energy treatment numbered fewer than the CFU released from the mock sample during the 30 min incubation (3- to 100-fold less), and were an insignificant fraction of the initial total biofilm CFU (*p* > 0.05, data not shown). Thus, as observed in Figure 4, the predominant mechanism of biofilm reduction by dual energy treatment was direct killing of the bacteria within the *P. acnes* biofilm.

It was of interest to know whether the synergistic bactericidal effect could be achieved by sequential rather than simultaneous exposure of biofilms to the two energies. Therefore, *P. acnes* biofilms were exposed to 15 min of light, followed by 15 min of ultrasound, or vice versa, with a 10 min rest between exposures. For comparison, biofilms were exposed to 15 min × 1, or 15 min × 2, of light plus ultrasound combined. Results indicated that delivering 15 min of light followed by a 10 min rest, then 15 min of ultrasound, or vice versa, yielded equivalent biofilm reduction. This was most likely due to the bactericidal effect of the light on the bacteria within the biofilms. The sequential application was less effective compared to delivering either a single 15 min exposure of the combination energies simultaneously, or two 15 min exposures of the combination (Table 3).

### 3.2. Kinetics of Biofilm Reduction and Regrowth after Exposure

**Figure 6:**
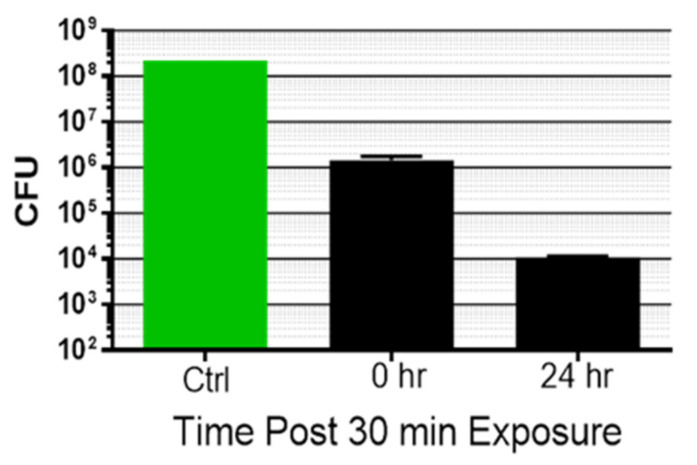
*P. acnes* biofilms ± exposure to simultaneous ultrasound plus light @ 80 mW/cm^2^ (144 J/cm^2^ for 30 min), with either immediate plating (0 h) or continued growth for 24 h, followed by plating. Bars are the mean ± std. dev., n ≥ 4, *p* < 0.0001 (ANOVA for differences of the mean) for the three determinations, *p* = 0.0082 (*t*-test for differences of the means) comparing the samples exposed at time 0 and harvested at time 0 versus exposed at time 0 and harvested 24 h post-exposure.

*P. acnes* biofilms were exposed a single time for 30 min to the combination of light and ultrasound, and either harvested immediately for CFU determination or returned to a fresh 6-well plate for continued culture. As previously observed, a single 30 min exposure led to an immediate reduction of bacteria within the biofilm (Figure 6). The CFU number harvested at 24 h post-exposure was further reduced. Thus, a single 30 min dual energy treatment induced declining biofilm CFU numbers over approximately 24 h.

**Figure 7:**
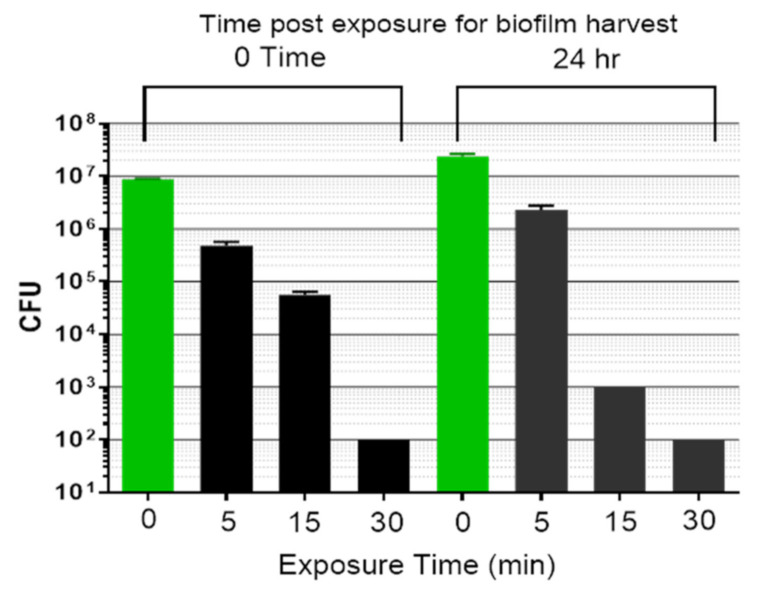
Comparison of CFU remaining in *P. acnes* biofilms either untreated (green bars) or after simultaneous energy treatment (ultrasound plus light@80 mW/cm^2^). CFU were determined immediately after exposure (black bars on left) or cultured for an additional 24 h after exposure (black bars on right). Data shown are the mean ± std. dev, n > 4, *p* < 0.0001 (ANOVA for differences of the mean) comparing the four determinations across means at 0 time after exposure, or at 24 h after exposure.

In subsequent experiments, additional exposure times were examined to determine what minimal dose of the dual energies could cause biofilm reduction lasting, or increasing, over 24 h. Biofilms were mock treated or exposed for 5, 15, or 30 min, and either harvested for CFU determination immediately (0 time post treatment, bars on left) or returned to 6-well culture plates for an additional 24 h incubation (bars on right) (Figure 7). After a 5 min exposure, there was an immediate drop in *P. acnes* biofilm CFU compared to the mock sample harvested at 0 time (~1 log_10_). When the 5 min exposed biofilm was incubated another 24 h and then harvested, the CFU number was reduced compared to the mock control harvested at 24 h (~1 log_10_), but the CFU did not continue to decrease compared to the 5 min sample harvested at 0 time. Following a 15 min exposure, the CFU count was immediately reduced compared to the mock sample harvested at 0 time (~2 log_10_), and this decline continued when the sample was incubated for an additional 24 h. After a 30 min treatment, there was a reduction in CFU to the lower limit of detection for the experiment (≤10^2^ CFU/mL) at 0 time, which persisted to the 24 h post-treatment point. Therefore, a 15–30 min treatment was sufficient to prevent biofilm regrowth for 24 h post-treatment.

**Figure 8:**
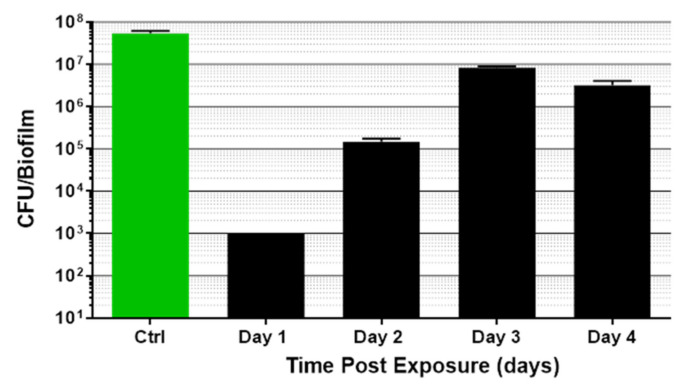
Time course of *P. acnes* biofilm death/regrowth after a single 30 min energy treatment with ultrasound and light (light @ 80 mW/cm^2^). Comparison of CFU remaining in *P. acnes* biofilms after exposure. Data shown are the mean (±std. dev) n > 4. *p* < 0.0001 (ANOVA for differences of the mean) of the five determinations, and *p* = 0.001 (*t*-test for differences of the mean) for Day 4 compared with Day 0 control.

To determine how long a single dual energy exposure of *P. acnes* would reduce the CFU number compared to an untreated control harvested at time 0, biofilms were exposed a single time (30 min) and returned to incubation at 37 °C until indicated harvest time (Figure 8). The viable biofilm remaining at 24 h post-treatment began to regrow after 48 h (Day 2). Compared to the mock control sample harvested at time 0, by 96 h post-treatment, *P. acnes* CFU number had nearly returned to the 0 time starting density; however, the CFU number remained significantly reduced compared to the 0 time untreated control (*p* = 0.001).

### 3.3. Comparison of Light Plus Ultrasound to Erythromycin for Biofilm Reduction

The previous experiments revealed the biocidal nature of combining light with low intensity ultrasound toward P. acnes biofilm, and the kinetics of death/regrowth after a single treatment. This energy combination was compared to an antibiotic, erythromycin (Fluka, 45703-10-F), against a *P. acnes* strain (6919) sensitive to erythromycin (MIC = 0.06 µg/mL) (data not shown) [28,32]. Biofilms were incubated with erythromycin for 24 h, harvested, and CFU determined. Alternatively, *P. acnes* biofilms were treated with light plus ultrasound for 5 or 30 min, and CFU was determined immediately after treatment.

**Figure 9:**
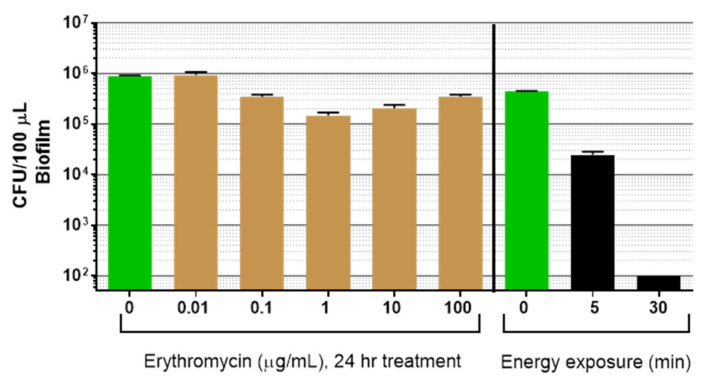
Dose–response curve of *P. acnes* biofilm treatment with erythromycin. Biofilms were either untreated (green bars) or treated for 24 h with the drug, then harvested and CFU enumerated for each treatment dose. Alternatively, *P. acnes* biofilms were exposed to a combination of light at 405 nm plus low intensity ultrasound for increasing exposure times, then biofilm was immediately harvested and CFU enumerated. Data shown are the mean (±std. dev), n > 4, for erythromycin *p* < 0.0001 (ANOVA for differences of the mean), and *p* < 0.0001 for dual energy exposure (ANOVA for differences of the mean).

Erythromycin significantly reduced the *P. acnes* biofilm (*p* < 0.0001), but the reduction was less than 1 log_10_ at the optimal concentration of 1 µg/mL (Figure 9). This is in general agreement with the reported antibiotic resistance of biofilm versus planktonic *P. acnes* [28]. In comparison, the combination energy exposure reduced *P. acnes* biofilms by ~1 log_10_ within 5 min (94%, *p* < 0.0001), and >3 log_10_ after 30 min treatment (>99.9%, *p* < 0.0001).

### 3.4. Treatment of Mammalian Cells with Simultaneous Light Plus Low Intensity Ultrasound

For the clinical application of light plus ultrasound, host cells should be relatively unaffected at energy levels which deliver significant bactericidal activity. Fibroblasts (murine 3T3 cells) and keratinocytes (primary, human) were tested for sensitivity to the combination treatment. 

3T3 cells were optimized for growth in hanging inserts. Cell inoculum number was titrated, and an optimal seeding concentration found to be 0.4–0.5 × 10^6^ cells/insert, which yielded 1.19 × 10^6^ ± 0.24 × 10^6^ cells/insert at 24 h. Cells appeared viable and healthy with Live/Dead staining. Conditions to enhance cell stability during exposure experiments included maintaining the temperature at 37 °C throughout the experiment and using DMEM with 10% FCS in the insert. These conditions enhanced the survival of mock treated samples; however, there was still a large variability of recovered cells after mock treatment. Therefore, to correct for cell numbers after mock or energy treatment, the percent cell viability (after trypan blue staining) is reported. Since exposure to light at 405 nm is a more potential safety concern for mammalian cells than exposure to low level ultrasound, light intensity was varied, while ultrasound was held constant (at 456 kHz@300 kPa). Cells were exposed as indicated in Table 4, and either harvested for viable cell counts after exposure or returned to the incubator for overnight growth. Due to the lack of sterility during the experiments and presence of nutrient rich medium favoring the outgrowth of contaminants, cells could not be cultured for longer than 24 h post-exposure without some contamination. Data shown are pooled from two independent experiments performed in duplicate.

Exposing 3T3 fibroblasts to ultrasound plus light, with light intensity ranging from 12 to 58 J/cm^2^, yielded cell viability and cell number values which fell within the range of the mock treated samples. There was a slight trend toward fewer cells with increasing light exposure at Day 1, but these values were no different than the range of values found among the mock samples, and the trend was not significant (*p* = 0.78, ANOVA for difference of the means).

Human primary keratinocytes were optimized for growth on the hanging inserts by comparing inoculation concentration, medium volumes, and time post passage of parent cultures prior to seeding of inserts. Trypsinization of keratinocytes on the hanging inserts did not give reliable quantification of cell number or viability; therefore, that method was abandoned. Optimal seeding was found at 4 × 10^4^ cells/insert in 4 mL total medium. Cultures were used at 48 h post seeding for experiments. Cells were used at passage number less than 9. Keratinocytes were exposed to ultrasound and light, in the same manner as the 3T3 cells, with increasing light intensity (and keeping total ultrasound output constant). After exposure, Alamar blue was added to the insert, with continued incubation at 37 °C and 5% CO_2_. Data shown are the range of results from two experiments performed in duplicate (Table 5).

**Figure 10:**
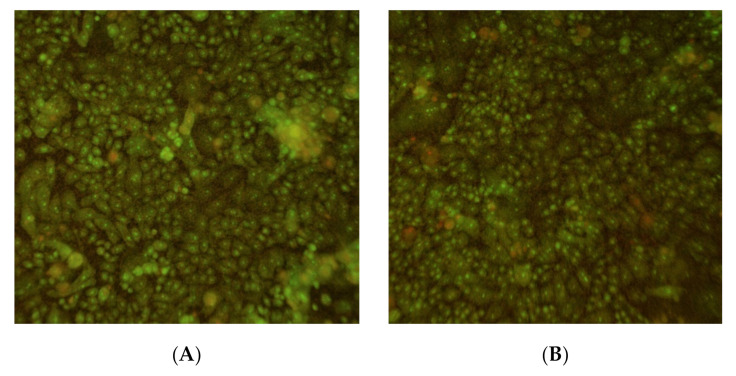
Live/Dead stain of human keratinocytes: (**A**) mock treated; (**B**) treated with light (58 J/cm^2^ light) plus ultrasound (**B**, right) at Day 1 post-treatment.

The percent reduction of Alamar blue is an indication of the metabolic activity of the keratinocytes; the higher the percent reduction (from an oxidized to a reduced form of the dye), the more metabolic activity is being exhibited by the test cells. The percent of Alamar blue reduction in cells exposed to light plus ultrasound at Day 0 was not different from that of the mock cells (*p* > 0.05), and slightly lower than the mock cells at Day 1, although this was only of borderline significance (*p* = 0.05, ANOVA for differences of the mean). Cells were also analyzed using Live/Dead BacLight stain after light plus ultrasound treatment (light at 58 J/cm^2^) in situ (Figure 10). No gross differences were observed between the mock and energy exposed cells at Day 1 post-exposure.

The results obtained after exposing mammalian cells to low intensity ultrasound simultaneously, with increasing blue light intensity, suggest that ultrasound with light energy to a maximum of 58 J/cm^2^ did not cause significant harm to these cells in vitro, up to 24 h after exposure. This is in comparison to the ≥10-fold reduction and >100-fold reduction of *P. acnes* biofilms exposed a single time to either 9 J/cm^2^ or 24–48 J/cm^2^, respectively, using the same in vitro test system.

## 4. Discussion

The key innovation of combining light and non-focused ultrasound stemmed from the realization that the combination of low, safe levels of light and ultrasound could act synergistically in a therapeutic manner, and that it was possible to combine these two energy types in a single treatment device. The next innovation was the application of this dual energy approach to reduce bacteria viability within biofilms.

Both ultrasound and light can, in some respects, affect bacteria and biofilms. Interaction of bacteria with each energy component relies on different molecular or physical entities; therefore, combining them should be complementary. Low intensity ultrasound can have dramatic but non-lethal effects on biofilms which render the bacteria more susceptible to antibiotics or chemical biocides [18,33,34,35,36]. Blue/violet light (400–470 nm) is directly bactericidal, most likely by interacting with porphyrins to produce singlet oxygen and hydroxide radicals [5,37,38,39] which lead to a loss of membrane integrity [7,31]. Although the biocidal mechanism of action for the combination of blue light and low intensity ultrasound is speculative, it appears that ultrasound’s mechanical stress on bacteria makes bacteria more susceptible to the biocidal nature of light, or, in some fashion, exacerbates the damage light causes.

To investigate the combination of low intensity ultrasound and light applied to bacteria in vitro, a device delivering both energies coincidentally was constructed. This device has a novel construction such that the ultrasound and light energy travel through an acrylic cone and impinge upon the same three-dimensional volume. A custom treatment chamber was engineered to accommodate treatment of biofilm grown in transwells. The transwell has a membrane made of PET upon which the biofilms were grown. Because the thin PET membrane is essentially transparent to ultrasound, the energy waves passed through the target cell layer and membrane, and were then absorbed by castor oil in the lower chamber. This prevented the ultrasound energy from reflecting back and provided for consistent ultrasound exposures, and also more closely simulated the exposure conditions found in-vivo. The exposure chamber system also allowed mammalian cells and planktonic bacteria to be treated with the same conditions as those used for the biofilms.

Planktonic *P. acnes* grown to stationary phase were not killed by low intensity ultrasound alone within the treatment period. This agrees with previous reports [40]. Light alone was bactericidal under these conditions but, with the addition of ultrasound, the bactericidal effect was significantly increased. This synergy was also observed when treating *P. acnes* biofilms. The combination energy treatment led to significant bacteria killing. This was demonstrated with Live/Dead viability staining of the biofilms, as well as biofilm CFU quantification. Maximum reduction of biofilm CFU was observed when both energies were delivered simultaneously, versus sequentially, to the biofilms. This indicates that the synergistic mechanism was dependent on short lived phenomena, which therefore implies that the two energies must be delivered concurrently. A similar finding was made by Pitt and coauthors, in which they saw the need for simultaneous low intensity ultrasound delivery with an antibiotic to increase antibiotic efficacy against planktonic and biofilm bacteria. In their work, low intensity ultrasound alone did not have biocidal action. It only enhanced antibiotic efficacy if delivered simultaneously with the antibiotic [41]. This would indicate a short-lived effect which quickly dissipates after removal of the ultrasound source.

Low intensity ultrasound could affect bacteria through either direct or indirect means. Bacteria are essentially transparent to ultrasound. That is, ultrasound waves pass through them with little absorption or interaction [42]. The mechanical effect of low intensity ultrasound pressure on bacteria is most likely related to its indirect effect on membrane integrity. Specifically, the passage of the ultrasound wave can create mechanical shear stress on the bacterial membrane. Further, ultrasound, even at low intensities, may induce stable cavitation on a microscale, which would amplify the shear stress effects. These hydrodynamic stresses may affect the permeability of the bacteria cell membrane, [42] which, in turn, could lead to an increase of oxygen within bacteria during energy exposure. An increased oxygen level would enhance killing mediated by light, as the production of toxic oxygen products through porphyrin interaction is dependent on cellular oxygen concentration [5,38]. Additionally, the mechanical stress effect on the bacteria could induce a biochemical stress effect, leading to the generation of intracellular nitric oxide [43]. Nitric oxide is a potent stimulator of biofilm dissemination, causing phenotypic changes in the bacteria [22]. The presence of NO could contribute to the biofilm disintegration observed with ultrasound alone. The number of CFU harvested ~24 h after treatment was found to be lower than the number of CFU harvested immediately after treatment. Continuing conversion of the bacteria within the biofilms to a non-adherent planktonic state could help explain this effect. Alternatively, creation of low levels of toxic products could initiate a slow cascade leading to eventual cell death of bacteria residing within the biofilm. The slow nature of the cascade might not interfere in outgrowth on nutrient agar until a critical level of cell damage occurred.

In terms of therapeutic implications of the results, one of the most widespread and important skin conditions in the United States is acne vulgaris, with approximately 8–10 million people afflicted with moderate to severe (inflammatory) acne. [44] Severe acne can lead to permanent facial scarring, depression, low self-esteem, and low quality of life for patients [44]. Importantly, current standard-of-care treatments for acne may be costly, require multiple doctor visits, have associated (serious) safety issues, and are not always effective [45,46,47]. The etiology of acne is multifactorial and is related to the presence of underlying *P. acnes* in pilosebaceous follicles [24,48]. Reduction or elimination of the bacteria leads to relief from inflammatory symptoms [28,49,50,51]. Treatment of acne vulgaris with blue light has been pursued but has not been widely adopted due to insufficient treatment efficacy [47]. The combination of ultrasound with light reported herein may positively affect acne treatment in vivo if the synergistic biocidal action observed in vitro can be replicated within the dermis. In comparison to a chemical agent used for acne treatment, erythromycin, the combination of energies was superior at killing *P. acnes* in a mature biofilm.

By keeping both energies at levels and wavelengths which have been shown to be, or are considered, safe (less than 100 mW/cm^2^ I_SATA.0_ for ultrasound and light outside of the ultraviolet range at 405–427 nm), the combination technology is expected to have minimal impact on normal dermal tissue. Although beneficial effects from individual use of both low intensity ultrasound and blue light have been reported in the scientific literature, safety must be a consideration for use of our dual energy exposure, since the two energies have not previously been combined in clinical use. The beneficial bioeffects of low power ultrasound include stimulation of extravasation of white cells, increased neutrophil antibacterial activity, and stimulation of collagen formation [52,53,54,55]. Known beneficial effects of blue light include transient stimulation of NO production (which leads to vasodilation in mammalian tissue), reduction of inflammation, and reduction of pro-inflammatory markers TNF-∞ and MMP-2 [56,57,58].

Blue light in the visible spectrum has been adopted for treatment of certain skin conditions, and safety associated with this clinical use has been investigated. The US Food and Drug Administration (FDA) has cleared several blue light devices for Over the Counter (OTC) use for acne and/or wrinkle treatment. Numerous studies have reported no harmful effects of blue light (405–420 nm) on adult human skin (including whole body treatment at 43.7 J/cm^2^ daily for 5 days, repeated 7 times) [59,60,61,62,63,64,65,66,67], reviewed in [5]. For patients undergoing whole body blue light treatment, no harmful effects on primary blood cells were observed, nor were depletion of dendritic cells from the dermis, DNA damage, or early photo-aging [59,60]. On a cellular level, work with blue light at 405 nm, up to 15 J/cm^2^, indicates no harm to in vitro cultured cells [56,62], nor does it induce genotypic changes [63]. However, there are also reports that blue light (400–450 nm) with fluence levels >66 J/cm^2^ may be detrimental to cultured skin cells (as determined metabolically by Alamar blue reduction) [64,65]. In investigations into the use of blue light (415 nm) for burn wound treatment, using a murine model, Zhang and co-authors determined that there was “no significant DNA damage detected in mouse skin by means of a skin TUNEL assay after a blue light exposure of 195 J/cm^2^” [66]. Although long term cancer risk from blue light cannot be absolutely ruled out, current evidence argues against it. Indeed, based on recent research, exposure to blue light increases pathways which are anti-inflammatory, antioxidative, and protect against cancer [67].

Using our experimental setup, exposure of mammalian cells to the dual energies of light plus ultrasound did not lead to gross disruption, decline, or death of the cells, and was analogous to mock treatment of the cells. Mammalian cells were resistant to harm from the exposure intensity level used to successfully treat *P. acnes* biofilms (>100-fold reduction). Although only 0–24 h post-exposure results could be collected, it is expected that major damage would be observed within this time frame, similar to that observed for in vitro energy treated biofilms.

The combination of blue light and low intensity ultrasound presents an innovative approach for treating *P. acnes* biofilms. Additional work indicates that the treatment is also biocidal toward *Staphylococcus aureus, Staphylococcus epidermidis*, and *Escherichia coli* biofilms (manuscript in preparation). Therefore, this approach may be a new method to eliminate clinical biofilms without the necessity of drugs.

## 5. Patents

The following patents relate to the work reported in this manuscript: 8,206,326, 8,574,174, 8,979,775, 9,498,650, 9,649,396, 10,207,125,1,0792,510.

## Figures and Tables

**Table 1:** Optimized biofilm growth conditions on hanging inserts.

Bacteria	Inoculum (CFU per Insert)	Adhering Medium	Adhering Time (h)	Growth Medium	Culture Time (Days)	Biofilm (CFU/Insert)
*P. acnes*, strain 6919	1–2 × 10^8^	DMEM/F10 1:1 (Dulbecco’s mod. Eagle’s med./Ham’s F10), Corning celgro	18–24	RCM (reinforced clostridial medium), Oxoid	4–7	10^7^–10^9^

CFU: Colony Forming Unit.

**Table 2:** Exposure parameters used to treat bacteria and mammalian cells.

	Frequency	Pressure	Energy	Intensity	Wavelength
Ultrasound	456 kHz	280 kPa		~80 mW/cm^2^_(Isata.0)_	3.3 mm
Light			9–144 J/cm^2^	30–100 mW/cm^2^	405 nm

**Table 3:** Comparison of CFU remaining in *P. acnes* biofilms after energy treatment.

Energy Treatment	Time	CFU *
Mock control	0	2.2 (0.73) × 10^8^
Light and ultrasound simultaneously	15 min	2.85 (0.24) × 10^5^
Light and ultrasound simultaneously	15 min, 15 min	9.2 (1.7) × 10^3^
Light alone then ultrasound alone	15 min, 15 min	2.8 (0.3) × 10^6^
Ultrasound alone then light alone	15 min, 15 min	2.6 (0.1) × 10^6^

*P. acnes* biofilms were exposed to light at 405 nm, @ 80 mW/cm^2^ plus low intensity ultrasound, or light followed by ultrasound, or vice versa, or no energy exposure (mock). * Data are the mean (std. dev), n > 2; *p* < 0.0001 (ANOVA for differences of the means), *p* < 0.0001 (*t* test for differences of the mean comparing #2 with #4 or #5).

**Table 4:** 3T3 cells mock treated or exposed to light plus ultrasound; cell viability measured immediately after exposure (Day 0) or 24 h later (Day 1).

Light Energy(J/cm^2^)	Recovered Cell Numbers Day 0	% Cell Viability Day 0	Recovered Cell Numbers Day 1	% Cell Viability Day 1
12	1.8–6.0 × 10^5^	95.6–96.6	0.7–1.0 × 10^6^	96.8–96.8
23	2.2–3.5 × 10^5^	90.9–95.0	0.7–0.8 × 10^6^	96.6–96.8
46	1.4–6.0 × 10^5^	89.3–93.3	0.5–0.7 × 10^6^	96.5–90.3
58	3.0–4.5 × 10^5^	88.4–91.1	0.4–0.6 × 10^6^	93.8–96.5
Mock	1.8–9.6 × 10^5^	95.1–98.0%	0.3–1.2 × 10^6^	94.3–97.7%

Data shown are the range of values for the experiment performed twice, in duplicate, *p* = 0.78 (ANOVA for differences of the mean among Day 1 results).

**Table 5:** Human keratinocytes mock treated or exposed to light plus ultrasound; cell viability measured immediately after exposure (Day 0) or 24 h later (Day 1)**.**

Light Energy (J/cm^2^)	Alamar Blue % Reduction Day 0	Alamar blue % Reduction Day 1
12	9–20%	19–26%
23	9–13%	17–21%
46	9–9%	15–22%
58	6–9%	30–30%
Mock	6–18%	29–33%

Data shown are the range of results for the experiment performed twice, in duplicate, *p* = 0.05, (ANOVA for differences of the mean among Day 1 results).

## Data Availability

Data available upon request.
